# Sub-2 mm Radial Arterial Access for Endovascular Embolization of an Idiopathic Intramammary Pseudoaneurysm: A Case Report

**DOI:** 10.7759/cureus.93532

**Published:** 2025-09-30

**Authors:** Sounak Paul, Srijit Saha, Sourav Tripathy, Avik Bhattacharyya, Anasua Chattopadhyay

**Affiliations:** 1 Interventional Radiology, CK Birla Hospitals, Calcutta Medical Research Institute, Kolkata, IND; 2 Radiodiagnosis, CK Birla Hospitals, Calcutta Medical Research Institute, Kolkata, IND

**Keywords:** barbeau c, embolization, interventional radiology, intramammary pseudoaneurysm, radial access

## Abstract

Radial arterial access is a relatively uncommon vascular access site. In the Indian subcontinent, the radial artery diameters are usually sub-2 mm. Here, we present a case of a spontaneous intramammary pseudoaneurysm (Im-PSA) arising from the left internal mammary artery, which was endovascularly treated via a sub-2 mm left radial access. Intermittent 50 µg nitroglycerine (NTG) was instilled every 15-20 minutes via the sheath, intraprocedurally, and a bolus of 100 µg NTG was instilled before a 1:1 sheath-catheter flush configuration. There was no intraprocedural radial artery spasm or postprocedural thrombosis. The preprocedural and intraprocedural steps have been outlined for ease of radial access in such a subset of patients. Im-PSAs are common after percutaneous breast biopsies, and spontaneous Im-PSAs are rare. We recommend that radial access via sub-2 mm radial arteries can be safely and successfully performed for subclavian or visceral arterial pathologies.

## Introduction

Idiopathic intramammary pseudoaneurysm (Im-PSA) is a rare entity. These usually arise from the internal mammary artery or its anterior intercostal branches. Two such cases have been reported according to a keyword search of [“idiopathic pseudoaneurysm” AND “breast”] and [“idiopathic pseudoaneurysm” AND “mammary”] [1,2].

Femoral arterial access has been the cornerstone of interventional radiology since its inception. Radial arterial access is a relatively newer route of entry into the vascular system [3]. In the Indian subcontinent, the average built is smaller than that of the Western population; hence, the radial artery diameters are usually lower than 2 mm.

In one study, the success rate of ultrasound-guided radial artery access was 90.6% [4]. Complications associated with radial access may occur, including dissection, perforation, occlusion, and bleeding with hematoma formation, which may lead to compartment syndrome [5].

We present a case of spontaneous Im-PSA from the left internal mammary artery (LIMA), which was endovascularly treated via a sub-2 mm left radial arterial access. There was no radial artery spasm or thrombosis. The procedural details have been outlined for ease of radial access in such a subset of patients.

## Case presentation

**Figure 1:**
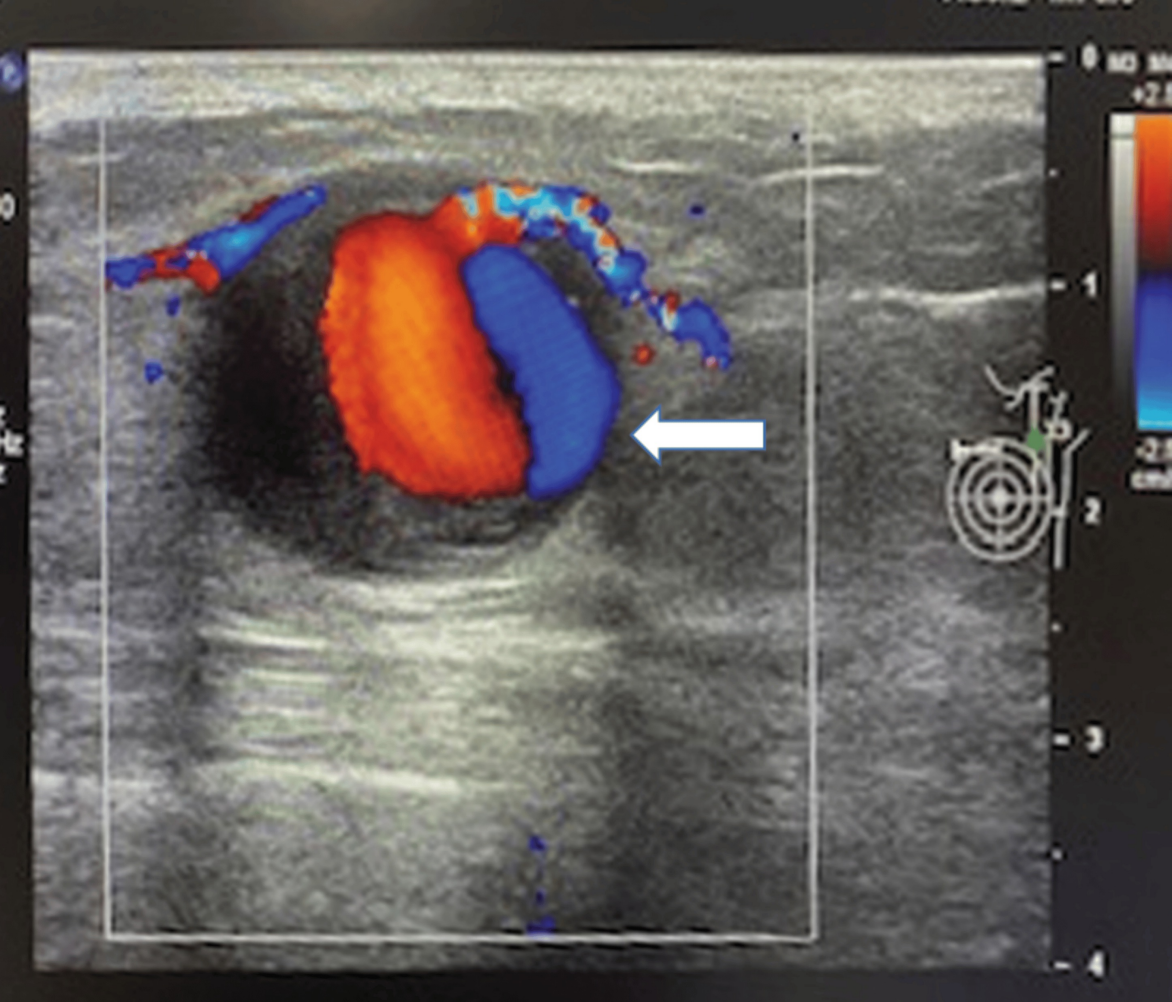
Doppler ultrasonography showing a pseudoaneurysm with a “yin-yan” flow pattern (white arrow) in the upper-inner quadrant of the left breast.

A 53-year-old woman presented with a left breast swelling and tenderness in the upper-inner quadrant. She was referred to the Department of Radiology for an image-guided core tissue biopsy. Ultrasound mammography revealed a 3.0 × 3.9 cm peripherally thrombosed pseudoaneurysm (Figure 1).

**Figure 2:**
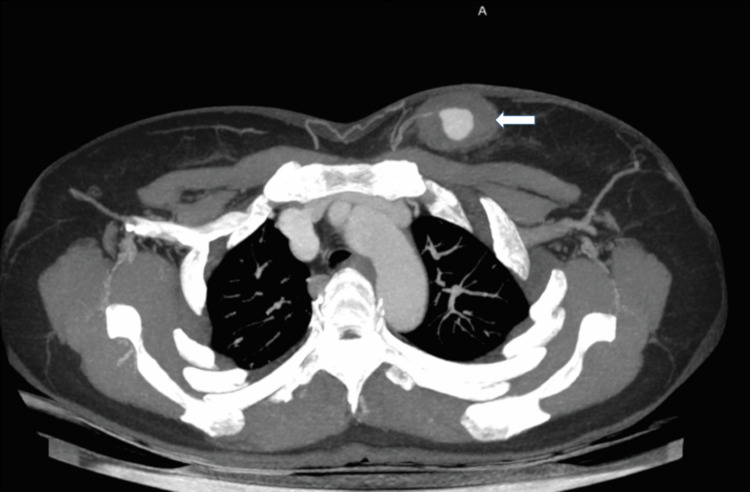
Axial maximum intensity projection post-contrast CT image illustrating a peripherally thrombosed pseudoaneurysm (white arrow) in the left breast.

**Figure 3:**
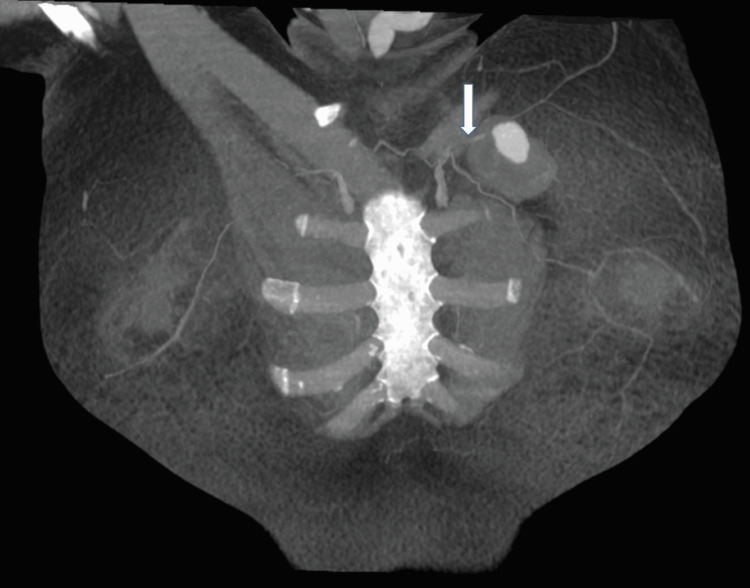
Coronal maximum intensity projection post-contrast CT image illustrating a branch of the left intermnal mammary artery (white arrow) leading into the left breast pseudoaneurysm.

CT angiography revealed multiple feeding arteries from the LIMA (Figures 2, 3).

Vascular access options included femoral or radial arterial access. The patient had a significant abdominal pannus covering the groin due to obesity, resulting in a hostile groin. Her left radial artery had a luminal diameter of 1.7 mm and a Barbeau type C spectral pattern on pulse oximeter [6]. In view of the pannus covering the groin and ease of postprocedural care in radial access, a left radial access was considered.

**Figure 4:**
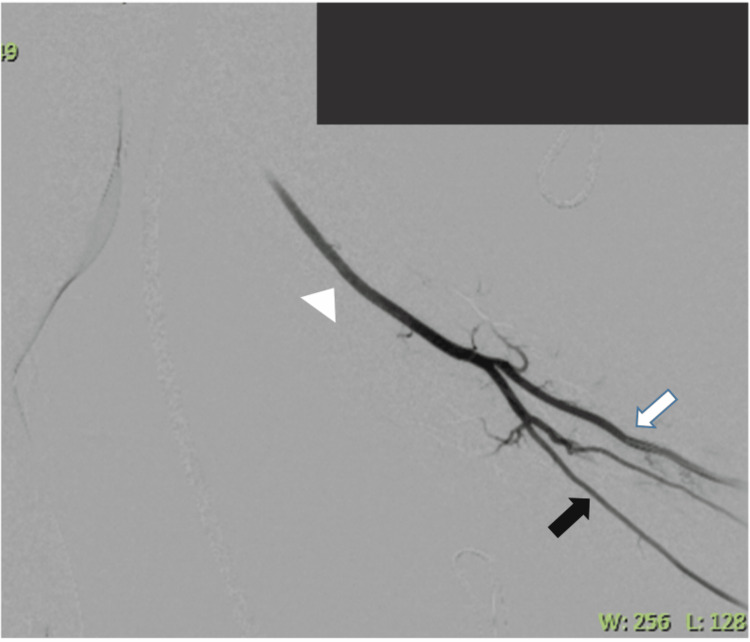
Left radial angiogram showing adequate left radial (white arrow), ulnar (black arrow), and brachial (white arrow-head) arteries. No vascular spasm and radial loop was noted.

Before arterial access, peri-arterial 50 µg nitroglycerine (NTG) and 1 mL bupivacaine were injected under ultrasound guidance. Lidocaine has been reported to cause vasospasm [7]. Hence, periarterial 50 µg NTG followed by 1 mg of bupivacaine was instilled [8]. A dwell time of one minute was allowed. This was followed by a single-wall puncture of the radial artery at the wrist, using a 4 Fr micropuncture set (Cook, Micropuncture Introducer Set, 4 Fr/10 cm) (Figure 4). The 3 Fr inner bit was exchanged for the needle over a 0.018” wire, and a standard vascular-cocktail (3,000 unfractionated heparin, 200 NTG, 2.5 mg diltiazem) was administered via this [9]. Thereafter, serial dilatation using the 3 Fr and 4 Fr dilators was telescoped together, followed by a 5 Fr radial sheath insertion over the 0.018” wire.

After sheath insertion, a 50 µg aliquot of NTG is instilled via the sheath and repeated every 15-20 minutes. The plasma half-life of NTG is 3 minutes, and by 15 minutes, nearly 95% of the drug is cleared from the circulation [10]. Intraprocedural radial artery was evaluated with ultrasound occasionally. The left supraclavicular region was prepared for a bail-out supraclavicular block in case of a radial artery spasm [11,12].

**Figure 5:**
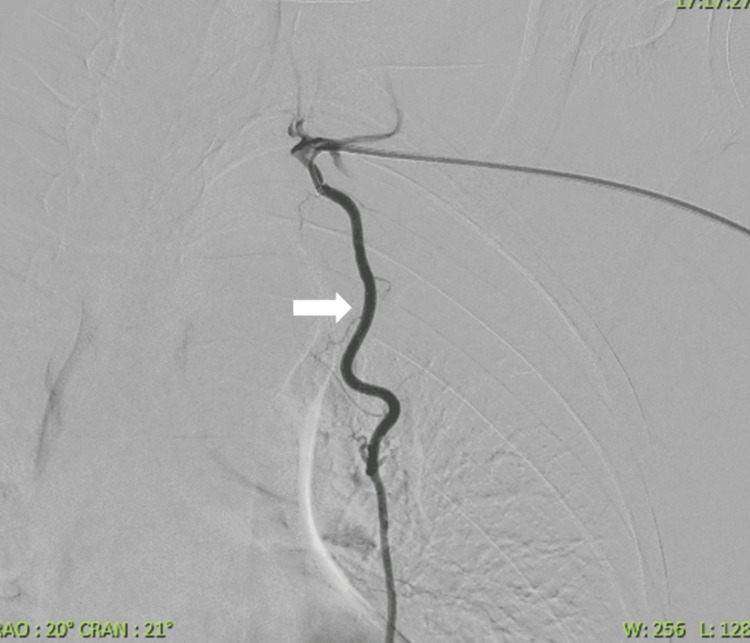
Early left internal mammary angiogram via a Rosch Inferior Mesenteric catheter showing a patent left internal mammary artery (white arrow) with a tortuous course.

For angiography and embolization, a 4 Fr, 120 cm long vertebral catheter was used to access the ipsilateral subclavian artery. A subclavian angiogram revealed a reverse curve configuration of the origin of the LIMA. Therefore, a 5 Fr 100 cm Rosch Inferior Mesenteric catheter was used to canulate the LIMA (Figure 5).

**Figure 6:**
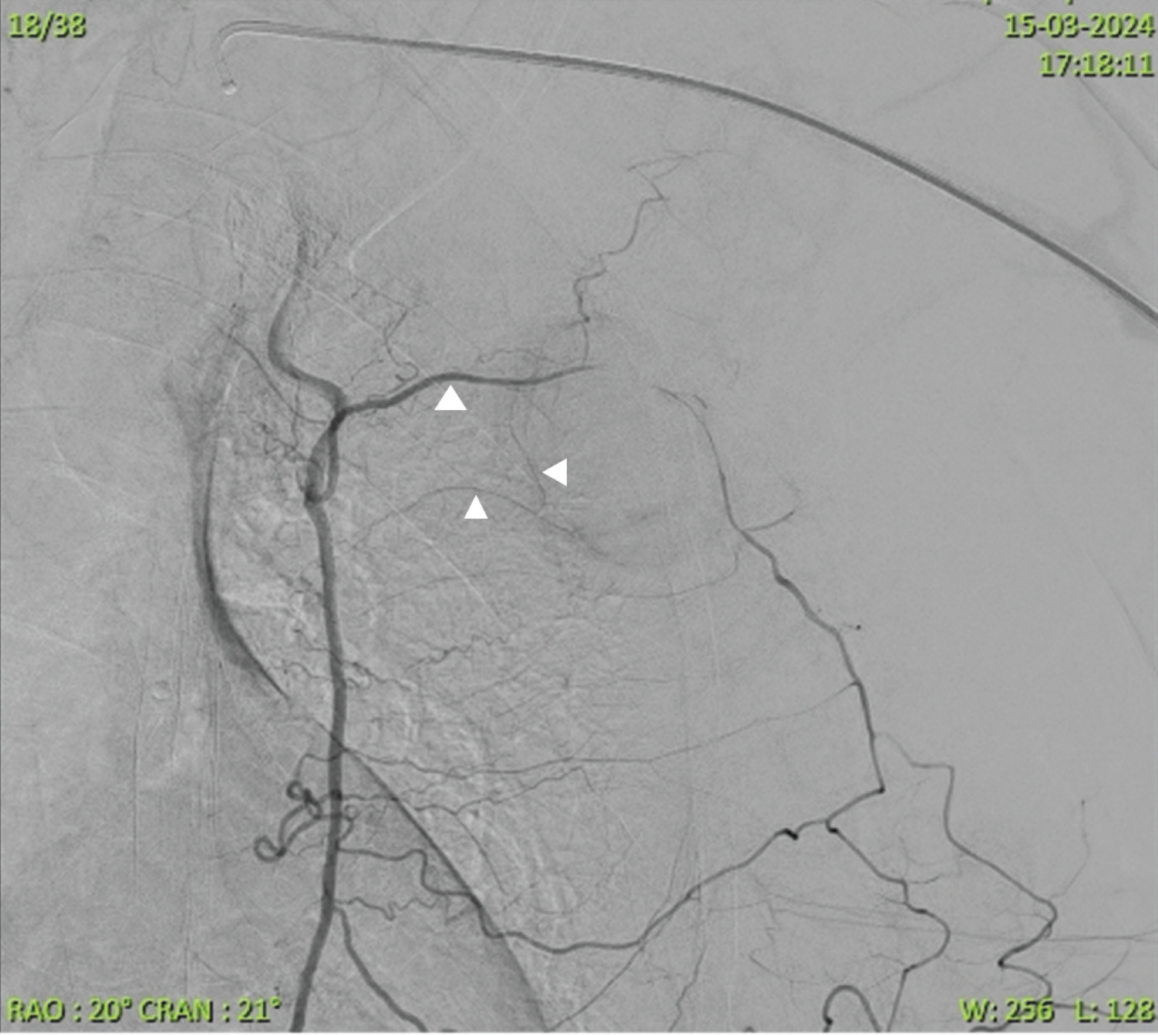
Delayed left internal mammary angiogram illustrating multiple irregular anterior intercostal arteries (white arrowhead) leading to the pseudoaneurysmal sac. The pseudoaneurysm is not seen due to vasospasm.

**Figure 7:**
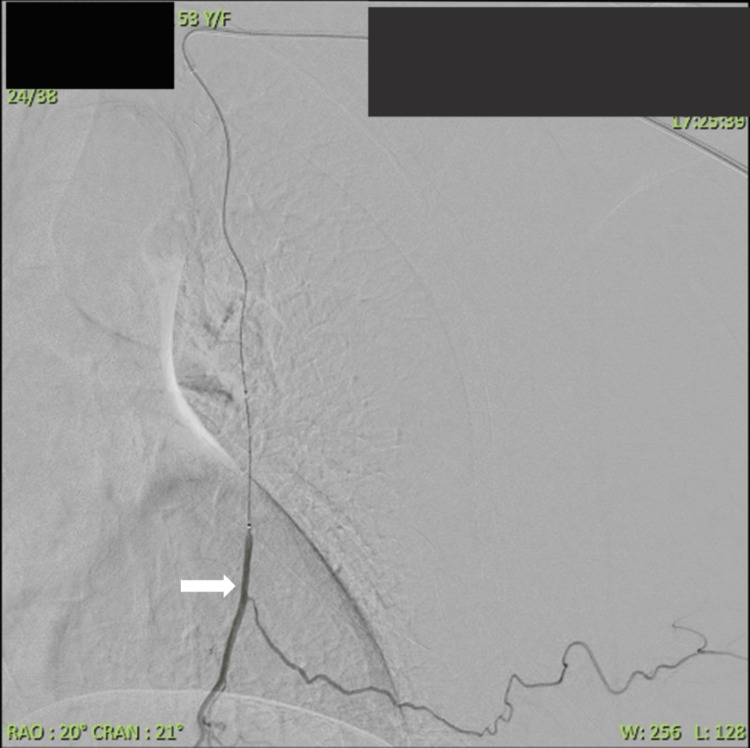
Distal left internal mammary angiogram showing a microcatheter negotiated into the distal left internal mammary artery (white arrow), caudal to the most distal anterior intercostal artery feeding the pseudoaneurysmal sac.

**Figure 8:**
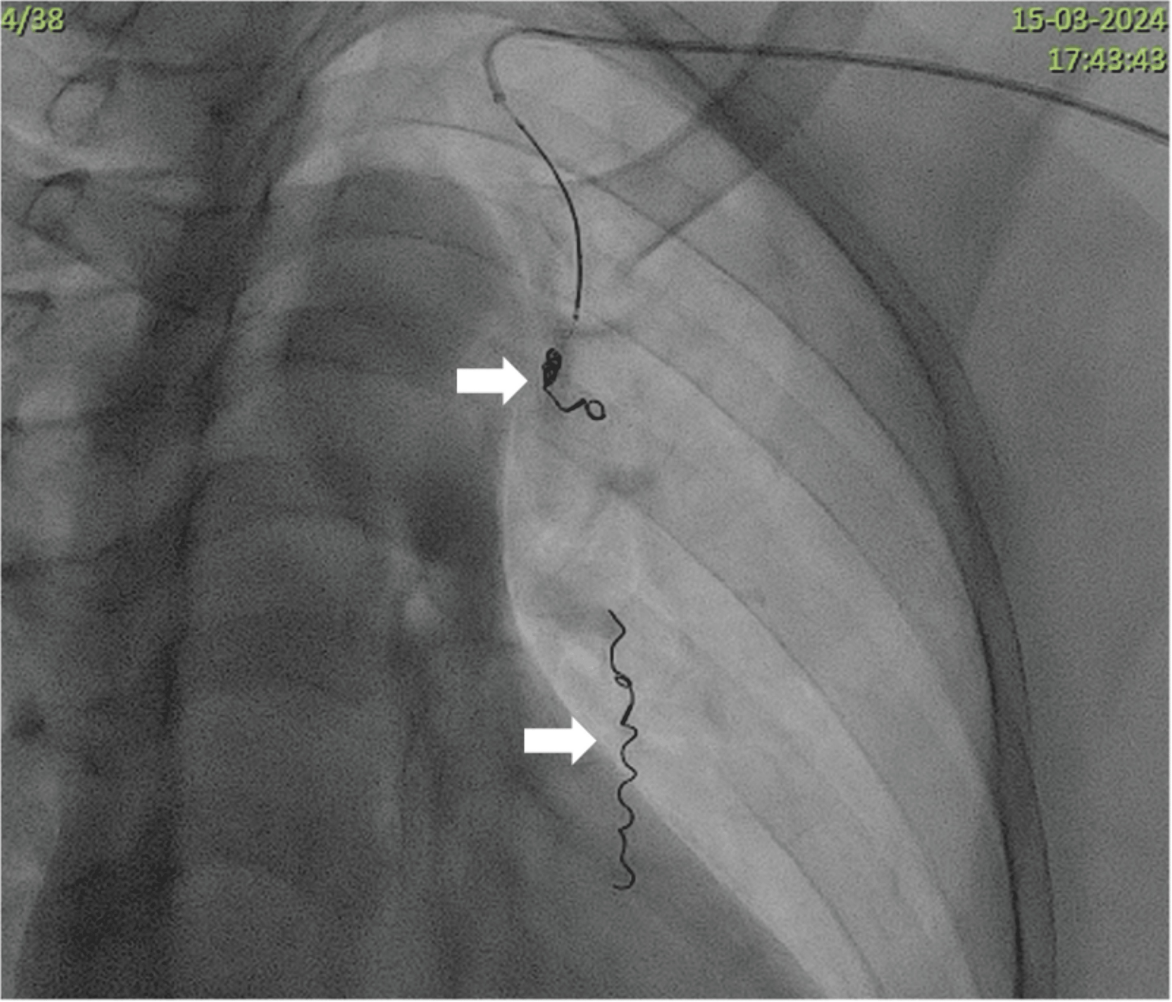
Coil embolization. The segment of the left internal mammary artery from which the feeders arise are embolized in a “sandwich technique” (white arrows) preventing distal reperfusion of the pseudoaneurysmal sac.

**Figure 9:**
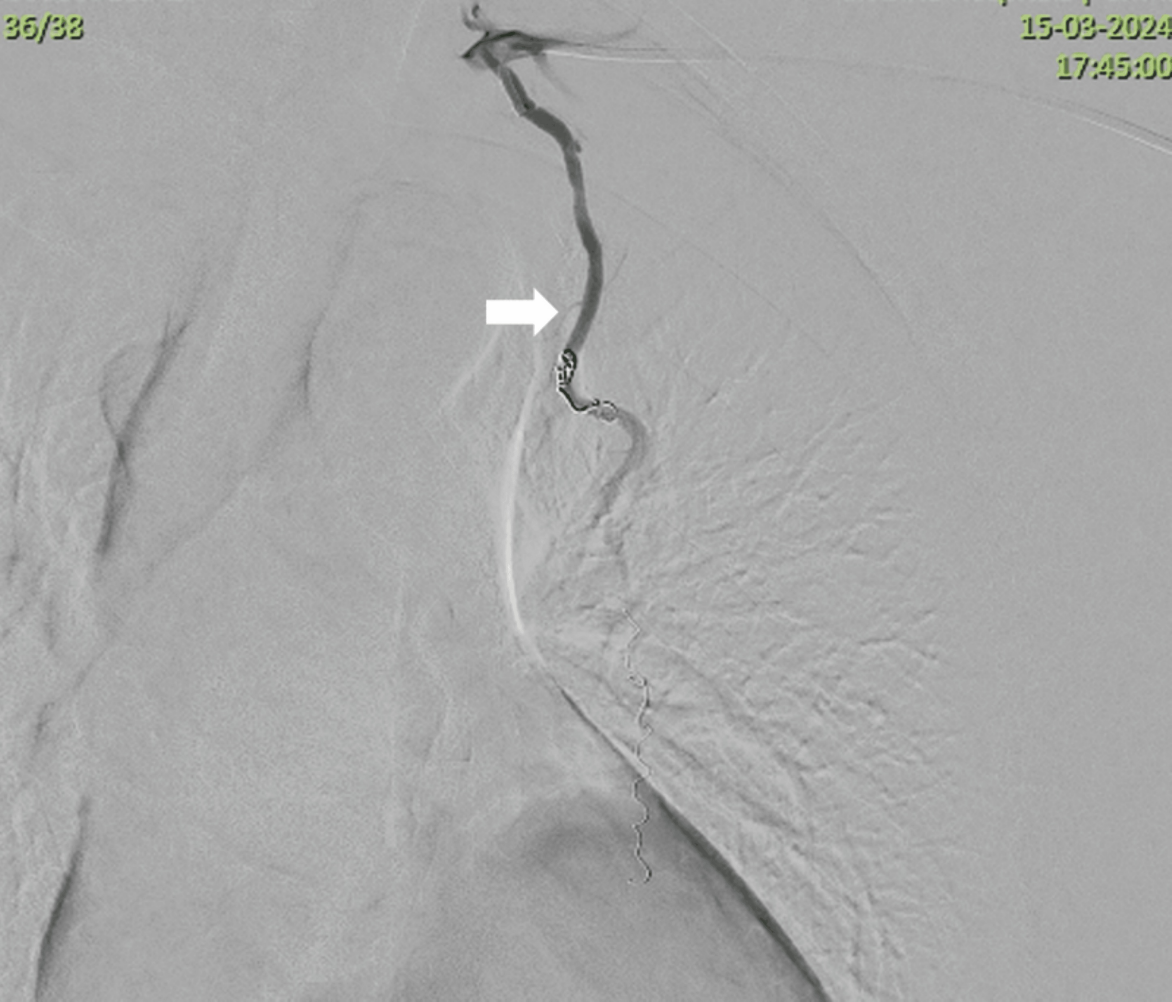
Completion angiogram showing stasis of flow in the left internal mammary artery (white arrow). No contrast filling-in of the pseudoaneurysmal sac can be seen.

**Figure 10:**
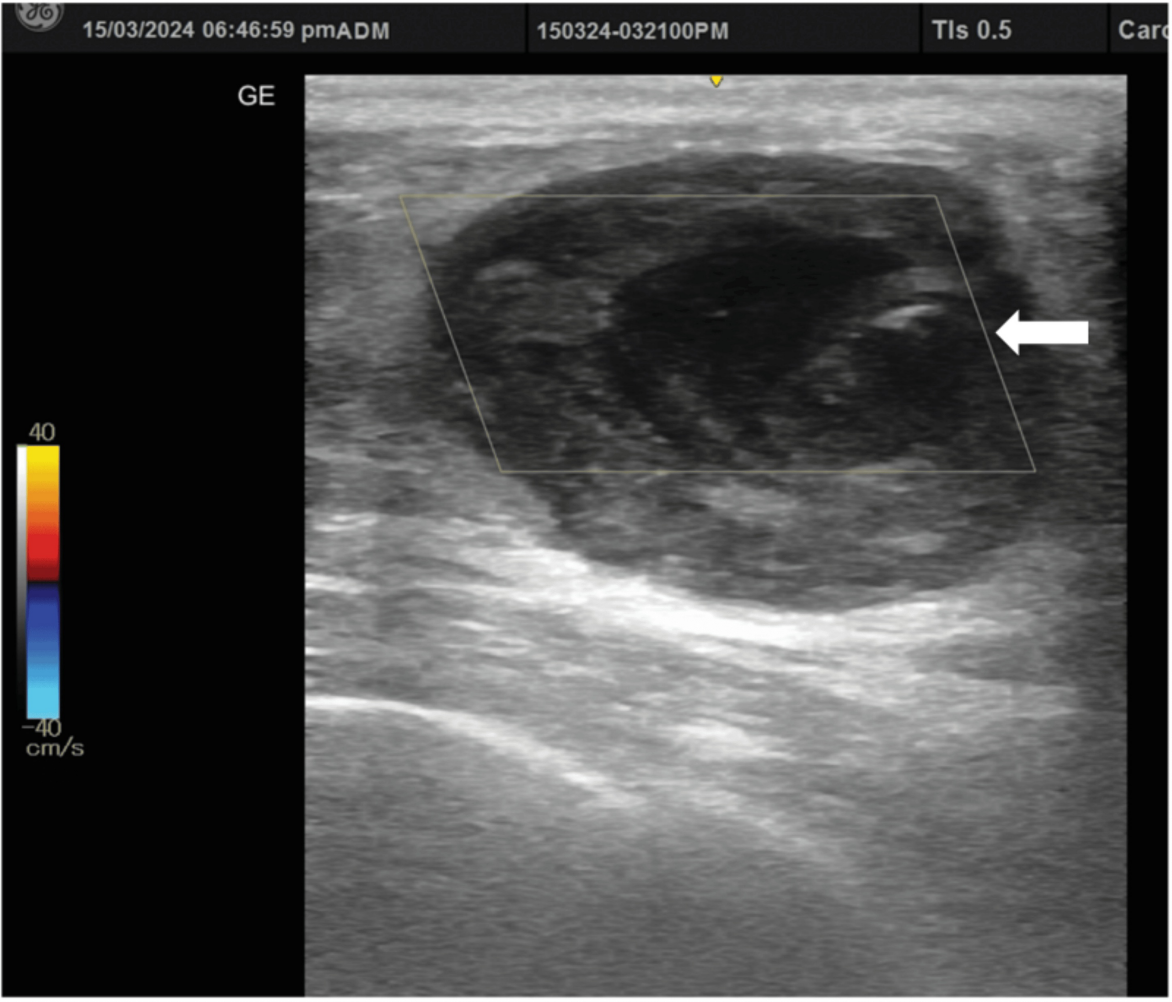
Doppler ultrasound showing thrombosis of the pseudoaneurysmal sac (white arrow). No postprocedure intra-sac Doppler flow can be noted.

Primary LIMA angiography revealed multiple feeders into the pseudoaneurysmal sac from the anterior intercostal branches (Figure 6). Distal access into the LIMA, distal to the supplying vessels leading to the Im-PSA for distal door occlusion (Figure 7). Thereafter, embolization was performed using 4 mm × 7 cm microcoils and 500-700 m PVA in a standard “sandwich-technique” (Figures 8, 9) [13]. Postprocedure Doppler evaluation revealed complete thrombosis of the pseudoaneurysmal sac (Figure 10).

Completion of the left radial artery Doppler revealed a patent left radial artery. The vascular sheath was removed, and hemostasis was achieved by manual compression.

## Discussion

Subcutaneous local anesthesia is usually achieved using 1% lidocaine for interventional radiological procedures. However, lidocaine has occasionally been associated with vascular spasm, increasing the difficulty of intraluminal access into a small artery. Hence, we used bupivacaine for local anaesthesia [7,8]. We presented a case of a spontaneous Im-PSA arising from the LIMA, which was endovascularly treated via a sub-2 mm left radial access. Intermittent 50 µg NTG was instilled every 15-20 minutes via the sheath following a bolus of 100 µg NTG.

Radial artery spasm occurs due to intimal damage during vascular access. A single wall puncture (preferably under ultrasound guidance) minimizes the intimal injury and reduces the chance of vascular spasm. Thereafter, administration of a pharmacological mixture of NTG and a calcium channel blocker reduces the chance of vascular spasm. Heparin reduces the chance of intraluminal thrombosis within the accessed vessel. This mixture induces pain during injection and is usually diluted with the blood, which is aspirated into the administering syringe before administering the mixture [9].

We recommend intermittent administration of NTG, as described, to prevent vascular spasm, which is occasionally expected in an outer diameter of sheath to inner vessel diameter ratio nearing 1:1. This will theoretically dilate the vessel and prevent spasm and thrombosis during the procedure. We have observed during our intraprocedural ultrasound screenings that there was no occasion of the vessel flushing the sheath, and that there was an intraprocedural patent lumen diameter of the target vessel, which was marginally more than the preprocedural inner vessel diameter.

This is a single case report, and is not a population statistic with limitations such as the lack of long‑term follow‑up or objective vessel diameter change measurements. However, we strongly believe that the results are reproducible, as we have had similar effects in a few of our prior interventions. However, a larger population-based study is recommended to identify the consistency of this result in a larger population.

Radial accesses for visceral interventions help in earlier patient mobilization and earlier hospital discharges, improving patient compliance and reducing chances of nosocomial infections.

## Conclusions

Im-PSAs are common after percutaneous breast biopsies. However, spontaneous or idiopathic Im-PSAs are rare and may be associated with underlying vasculitis or fibromuscular dysplasia. Before percutaneous biopsy of any breast lesion, it is imperative to evaluate the vascularity of the lesion by Doppler ultrasound. In doubtful cases, a contrast-enhanced CT of the thorax or post-contrast MRI of the breast may be obtained for adequate evaluation before image-guided sampling. Radial access can be safely done in sub-2 mm radial arteries. To avoid periprocedural complications, instillation of NTG may be considered in the peri-arterial tissue and intermittently via the sheath during the procedure. We recommend that radial access in sub-2 mm radial arteries can be safely and successfully performed for subclavian or branch arterial pathologies.
